# Three stages of laboratory stewardship in improving appropriate *Clostridioides difficile* testing in a community-based setting

**DOI:** 10.1017/ash.2025.55

**Published:** 2025-03-19

**Authors:** Michael S. Wang, Gretchen Zimmerman, Theres Klein, Bethany Stibbe, Monica Rykse, Samuel Ballard, Naveen Vijayam, Joe Brown, Khateeb Raza, Shannon Beckman, Andrew M. Skinner

**Affiliations:** 1 Osteopathic Medical Specialties, Michigan State University College of Osteopathic Medicine, East Lansing, MI, USA; 2 Department of Medicine, Corewell Health Southwest, Saint Joseph, MI, USA; 3 Department of Medicine, Central Michigan University School of Medicine, Saginaw, MI, USA; 4 Division of Infectious Diseases, University of Utah School of Medicine, Salt Lake City, UT, USA; 5 Research and Infectious Diseases Section, George E. Wahlen VA Medical Center, Salt Lake City, UT, USA

## Abstract

**Objective:**

Assess the efficacy of staged interventions aimed to reduce inappropriate *Clostridioides difficile* testing and hospital-onset *C. difficile* infection (HO-CDI) rates.

**Design:**

Interrupted time series.

**Setting:**

Community-based.

**Methods/Interventions:**

National Healthcare Safety Network (NHSN) *C. difficile* metrics from January 2019 to November 2022 were analyzed after three interventions at a community-based healthcare system. Interventions included: (1) an electronic medical record (EMR) based hard stop requiring confirming ≥3 loose or liquid stools over 24 h, (2) an infectious diseases (ID) review and approval of testing >3 days of hospital admission, and (3) an infection control practitioner (ICP) reviews combined with switching to a reverse two-tiered clinical testing algorithm.

**Results:**

After all interventions, the number of *C. difficile* tests per 1,000 patient-days (PD) and HO-CDI cases per 10,000 PD decreased from 20.53 to 6.92 and 9.80 to 0.20, respectively. The EMR hard stop resulted in a (28%) reduction in the CDI testing rate (adjusted incidence rate ratio ((aIRR): 0.72; 95% confidence interval [CI], 0.53 to 0.96)) and ID review resulted in a (42%) reduction in the CDI testing rate (aIRR: 0.58; 95% CI, 0.42–0.79). Changing to the reverse testing algorithm reduced reported HO-CDI rate by (95%) (cIRR: 0.05; 95% CI; 0.01–0.40).

**Conclusions:**

Staged interventions aimed at improving diagnostic stewardship were effective in overall reducing CDI testing in a community healthcare system.

## Introduction

*Clostridioides difficile* infections (CDI) cause significant morbidity to patients and place a significant financial burden on healthcare systems.^1,2^ Specifically, CDI is the leading cause of healthcare-associated diarrhea in the United States and contributes at least $4 billion annually in additional healthcare costs in the United States.^1,3^ As CDIs have primarily been viewed as a healthcare-associated infection, institutions have implemented numerous strategies to decrease hospital-onset CDI (HO-CDI).^4–6^

Since their approval in 2009, clinical laboratories have increasingly used nucleic acid amplification test (NAAT) for the detection of *C. difficile.* However, due to the increased sensitivity of NAAT, the incidence of *C. difficile* rose by nearly (70%) within the US.^7^ Thus, CDI subsequently became a target quality metric for the Centers for Medicare & Medicaid Services (CMS) value-based incentive programs in October 2016 with the aim to decrease HO-CDI.^8,9^

In 2017 the Infectious Diseases Society of America and Society for Healthcare Epidemiology of America CDI guidelines recommended a 2-step algorithmic approach for the detection of *C. difficile.* ^10^ These guidelines recommended the use of a sensitive test such as glutamate dehydrogenase or NAAT within the appropriate clinical context, followed by a more specific *C. difficile* toxin enzyme immunoassay (EIA).^10^ These recommendations aimed to provide another data point for healthcare practitioners in discriminating between *C. difficile* colonization and true CDI given the concern that these highly sensitive tests could not discriminate between true infection and colonization.^10–12^ As such, healthcare facilities have devised methods aimed at reducing inappropriate *C. difficile* testing with the goal of decreasing the overall prevalence of CDI and the incidence of HO-CDI.^13–15^ However, there is a paucity of data exploring how these interventions impact community-based healthcare facilities which typically have limited resources. At our institution, we implemented multiple interventions over a four-year period to improve *C. difficile* testing stewardship.

## Methods

### Study design and population

The study was a retrospective observational study of inpatients that took place at two community-based acute care hospitals located in St Joseph, Michigan and Niles, Michigan, assessing the impact of the three separate infection control and laboratory interventions. These interventions were enacted to address the rising number of documented HO-CDI cases which were thought to stem from inappropriate *C. difficile* testing.

The primary hospital within the hospital system is a 220-bed full-service community hospital and the secondary hospital is a 76-bed hospital with limited services. Both hospitals are reported as a single institution to the National Healthcare Safety Network (NHSN). The hospital system has 3 infectious diseases (ID) physicians, 4–5 infection control practitioners (ICP) during the study, a microbiology laboratory that conducted *C. difficile* testing at the primary hospital, and an antimicrobial stewardship pharmacist. Interventions at both hospitals were enacted at the same time and overseen by the group of providers previously noted. Data was collected from January 1, 2019, to November 30, 2022. The study was reviewed by the Corewell Health Institution Review Board and deemed exempt (IRB no. 1648).

### First intervention: electronic medical record (EMR) intervention

The first intervention was implemented in October 2019 with the stated aim of reducing the number of inappropriate *C. difficile* tests completed. After key stakeholders within the hospital were notified of potential financial penalties associated with an elevated HO-CDI rate. Hospital executives approached the ID group prior to the first intervention with the stated goal of reducing HO-CDI rates. The infection control practitioners (ICP) crafted a “hard stop” order that would require ordering providers to acknowledge that a patient had ≥3 loose watery stools (Supplemental Figure 1) over 24 h without laxative use prior to ordering a *C. difficile* test (Supplemental Figure 2). The order was approved by ID staff and the hospitalist group and deployed into the EMR.

### Second intervention: ID intervention

The second intervention was implemented in August 2020 after hospital executives were notified the hospital remained at risk of further financial penalties associated with elevated HO-CDI rates. The ID group was approached at how to further curb rates and the group elected to review all orders that had been placed >3 days after hospitalization with the explicit goal of reducing inappropriate testing in situations where a positive test result would result in a HO-CDI case. Orders were reviewed by the on-call ID physician who was available to review cases 24h a day, 7 days a week. To ensure compliance, ICPs crafted an order in the EMR that would display when a provider ordered a *C. difficile* test in a patient who had been admitted for >3 days with instructions to reports the stool frequency, laxative use, and to contact the on-call ID physician to approve the order (Supplemental Figure 3). After approval of the new EMR order, they were deployed to both hospitals.

### Third intervention: infection control and change in testing intervention

The third intervention measured was multifaceted and was initiated in March 2022 with the goal of further reducing the HO-CDI case burden within the hospitals. The first component of this intervention consisted of a change in the reported laboratory detection of *C. difficile* by implementing a multistep testing algorithm.

From January 2019 to February 2022 *C. difficile* was detected by NAAT (Xpert *C. difficile*, Cepheid, Sunnyvale, CA). Starting in March 2022, initial *C. difficile* detection was completed by NAAT, and positive NAAT tests were confirmed by toxin EIA (TechLab, *C. diff* Quick Chek Complete, Blacksburg, VA). By changing to the testing algorithm, we anticipated a reduction in the total number of CDI cases reported to NHSN due to the decreased sensitivity but increased specificity of toxin EIA testing.

The addition of a new test required the microbiology lab to ensure they had the necessary resources and staff to add a new test. The institution elected to report the NAAT and Toxin EIA tests separately to the ordering provider. After internal review by the laboratory and confirmation that testing would only be completed on liquid stool specimens, the test was implemented.

The other facet of the third intervention included ICP staff reviewing all inpatient *C. difficile* orders. On weekdays, ICPs would review a daily list of *C. difficile* orders in hospitalized patients. If there was an order for a patient that had been hospitalized for >3 days, they would determine if the patient had ≥3 loose stools over a 24 h period without laxative exposure. If these criteria were met, they would contact the on-call ID physician to approve the order described in the second intervention. However, if symptoms were not present, they would inform the ordering provider of why the test was denied. Additionally, the ICP would contact the nursing staff to collect a stool specimen for any *C. difficile* test ordered on day 3 to ensure testing was done prior to the 3-day deadline, which could result in an inappropriate HO-CDI classification.

### Study outcomes and definitions

The primary study outcomes were the incidence of completed and processed *C. difficile* tests per 1,000 patient days (PD) prior to all interventions and after each subsequent intervention. Our secondary outcomes were the: (1) percent test positivity, (2) incidence rate of HO-CDI per 10,000 PD, (3) admission CDI prevalence per 100 admissions, and (4) total CDI prevalence per 100 admissions as defined by NHSN.^16^

### Statistical analysis

The incidence rate (IR), percent positive, and prevalence rate (PR) were reported for the primary and secondary outcomes as appropriate. The crude incidence rate ratio (cIRR) was determined for the number of *C. difficile* test per 1,000 PD and HO-CDI cases per 10,000 PD with (95%) confidence intervals (CI). The crude prevalence rate ratio (cPRR) was reported for the admission and total CDI prevalence per 100 admissions with (95% CIs).

A negative binomial interrupted time series analysis was performed to determine the adjusted IRR (aIRR) with (95% CIs) to determine the impact that each intervention had on *C. difficile* testing per 1,000 PD as well as the monthly change after each intervention was implemented. The model was adjusted for the months prior to all interventions, and a separate intervention variable for each intervention, and a time-since-intervention variable were used for each intervention to determine the post-intervention impact. Additional variables included in the final model were a COVID-19 variable to account for the potential impact of COVID-19 on *C. difficile* and the average monthly length of hospitalization to account for the increased odds of having a *C. difficile* test due to prolonged hospitalization.^17^ Refer to the supplemental document for full model construction methods.

The *χ* ^2^-test and the Fisher exact test were used to compare categorical variables where appropriate. The normality of continuous variables was determined by the Shapiro–Wilk method. Student’s *t*-test was used to compare parametric variables. A *P*-value of ≤0.05 was considered significant and all tests were two-tailed. All statistical analysis was completed using R v4.3.1.

## Results

From July 2019 to November 2022, there were a total of 2,507 *C. difficile* NAAT tests completed in the inpatient setting of which (17.3%, 435/2,507) tested positive. Most *C. difficile* tests were completed between hospital days 1 and 3 (68.5%, 1,714/2,507) and (19.0%, 326/1,714) were positive for *C. difficile* by NAAT. Among tests completed after hospital day 3, (13.8%, 109/793) were positive by NAAT (Table 1). The average monthly length of hospitalization for the entire study was (5.12 days [95% CI, 4.94–5.31]).

**Table 1.** *Hospital and Clostridioides difficile* testing characteristics

	Total study	Pre-intervention period	Intervention 1 period	P-value for pre-intervention to intervention 1	Intervention 2 period	P-value for intervention 1 to intervention 2	Intervention 3 period	P-value for intervention 2 to intervention 3
Average length of hospitalization (95% CI)table	5.12 (4.94–5.31)	4.39 (4.29–4.49)	4.65 (4.52–4.79)	<0.01	5.39 (5.19–5.59)	<0.01	5.80 (5.42–6.19)	0.05
Proportion of positive *C. difficile* tests (%)	435/2507 (17.4%)	135/859 (15.7%)	82/535 (15.3%)	0.87	152/767 (19.8%)	0.04	66/346 (19.1%)^‡^/17/346 (4.9%)^†^	0.77^‡^/<0.01^†^
Proportion of *C. difficile* tests completed on hospital days 1–3 (%)	1714/2507 (68.4%)	559/859 (65.1%)	371/535 (69.3%)	0.10	515/767 (67.1%)	0.40^c^	269/346 (77.7%)	<0.01
Test positivity of *C. difficile* tests completed on hospital days 1–3 (%)	326/1714 (19.0%)	94/559 (16.8%)	59/371 (15.9%)	0.71	114/515 (22.1%)	0.03	54/269 (21.9%)^‡^/16/269 (6.0%)^†^	0.50^‡^/<0.01^†^
Test positivity of *C. difficile* tests completed after hospital day 3 (%)	109/793 (13.8%)	41/300 (13.7%)	23/164 (14.0%)	0.91	38/252 (15.1%)	0.77	7/77 (9.1%)^‡^/1/77 (0.4%)^†^	0.18^‡^/<0.01^†^

*Pre-intervention period:* January 2019–September 2019.

*Intervention 1 period:* October 2019–July 2020.

*Intervention 2 period:* August 2020–February 2022.

*Intervention 3 period:* March 2022–November 2022.

‡ NAAT positive only.

† NAAT positive/toxin enzyme immunoassay positive.

**Table 2.** *Clostridioides difficile* infection National Healthcare Safety Network (NHSN) metrics across baseline and intervention periods

CDI Metric	Pre-intervention period	Intervention 1 period	Pre-intervention to intervention 1	Intervention 2 period	Intervention 1 to intervention 2	Intervention 3 period	Intervention 2 to intervention 3
Total C. *difficile* testing incidence per 1,000 patient-days (95% CI)	20.53^a^ (19.18–21.95)	11.01^a^ (10.10–11.99)	0.54^b^ (0.48–0.60)	7.45^a^ (6.93–7.99)	0.68^b^ (0.61–0.75)	6.92^a^ (6.21–7.69)	0.93^b^ (0.82–1.05)
HO-CDI incidence per 10,000 patient-days (95% CI)	9.80^a^ (7.03–13.29)	4.74^a^ (3.00–7.11)	0.48^b^ (0.29–0.81)	3.69^a^ (2.61–5.07)	0.78^b^ (0.46–1.31)	1.40^a‡^ (0.56–2.88)/0.20^a†^ (0.01–1.11)	0.38^b‡^ (0.17–0.85)/0.05^b†^ (0.01–0.40)
Admission CDI prevalence per 100 admissions (95% CI)	0.98^d^ (0.80–1.20)	0.56^d^ (0.43–0.73)	0.57^e^ (0.41–0.79)	0.60^d^ (0.49–0.72)	1.05^e^ (0.77–1.45)	0.68^d‡^ (0.52–0.88)/0.19^d†^ (0.11–0.30)	1.15^e‡^ (0.84–1.57)/0.31^e†^ (0.18–0.52)
Total CDI prevalence per 100 admissions (95% CI)	1.41^d^ (1.18–1.67)	0.78^d^ (0.62–0.97)	0.55^e^ (0.42–0.73)	0.79^d^ (0.67–0.93)	1.01^e^ (0.78–1.33)	0.76^d‡^ (0.59–0.97)/0.20^d†^ (0.11–0.32)	0.96^e‡^ (0.72–1.28)/0.25^e†^ (0.15–0.41)

*Pre-intervention period*: January 2019–September 2019.

*Intervention 1 period*: October 2019–July 2020.

*Intervention 2 period*: August 2020–February 2022.

*Intervention 3 period*: March 2022–November 2022.

^a^Incidence rate.

^b^Crude incidence rate ratio.

^c^*P*-value.

^d^Prevalence rate.

^e^Crude prevalence rate ratio.

‡ NAAT positive only.

† NAAT positive/toxin enzyme immunoassay positive.

NHSN: National Healthcare Safety Network; CDI: *Clostridioides difficile* infection; HO-CDI: Healthcare-onset CDI; CI: Confidence interval.

### Pre-intervention

From January 2019 to September 2019 there were 20.53 *C. difficile* tests completed per 1,000 PD (95% CI, 19.18–21.95) with a test positivity of (15.7%, 135/859). Most tests, (65.1%, 556/859), were completed between hospital day 1 and 3, of which, (16.8%, 94/559) were positive. Prior to any interventions, the rate of *C. difficile* tests completed was decreasing by (4%) per month (aIRR 0.96, [95% CI, 0.92–0.99], *P* = 0.02).

### First intervention

From October 2019 to July 2020, the number of *C. difficile* tests decreased to (11.01 [95% CI, 10.10–11.99]) per 1,000 patient days. The unadjusted *C. difficile* testing incidence decreased by (46%) (cIRR: 0.54, 95% CI, 0.48–0.60) and after adjustment, the intervention attributed in a (28%) reduction in *C. difficile* test completed per 1,000 PD (aIRR: 0.72; 95% CI, 0.53–0.96). However, the months following initiation of the first intervention attributed to a (6%) increase in testing per month (aIRR: 1.06; 95% CI, 1.00–1.13).

**Figure 1.**
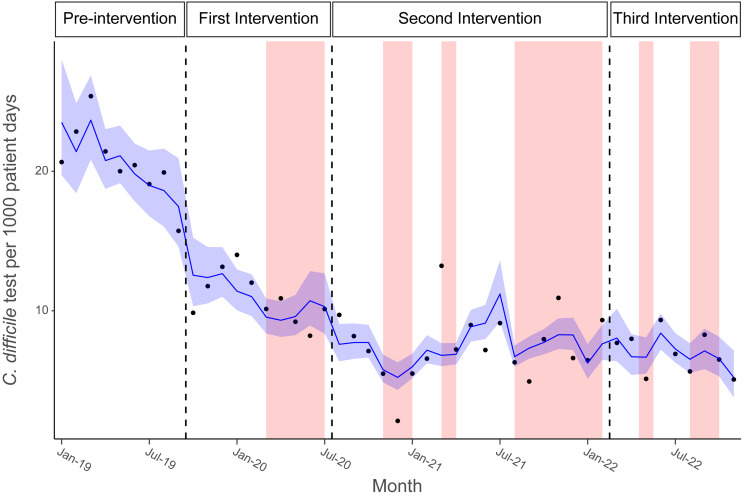
Interrupted time series (ITS) of completed *C. difficile* tests per 1,000 patient days, January 2019–November 2023. Each point represents the *C. difficile* test per 1,000 patient days for a single month. Thick blue line represents modeled *C. difficile* test per 1,000 patient days for each timeframe with (95%) confidence intervals. Interventions are depicted horizontally by a black dashed line. The red-shaded regions indicate a COVID-19 wave present in Michigan defined as≥30 admissions per 100,000 people. Pre-intervention: January 2019–September 2019; First intervention: October 2019–July 2020; Second intervention: August 2020–February 2022; Third intervention: March 2022–November 2022.

While there was a downward trend in the number of *C. difficile* test incidences during this time (Figure 1), this was likely due to COVID-19. COVID-19 waves were accountable for a (21%) reduction in *C. difficile* testing rate (aIRR: 0.79; 95% CI, 0.67–0.92) as well as a (39%) reduction in the reported admission CDI prevalence rate (aIRR: 0.61; 95% CI, 0.42–0.90) and a (31%) reduction in the reported total CDI prevalence rate (aIRR: 0.69; 95% CI, 0.49–0.94) (Table 3, Supplemental Tables 2 and 3).

**Table 3.** Adjusted incidence rate ratio of *Clostridioides difficile* test completed per 1000 patient days

	Immediate impact on IRR (95% CI)	*P*-value	IRR change per month (95% CI)	*P*-value
Pre-intervention			0.97 (0.92–1.01)	0.12
1^st^ intervention: Electronic medical record hard stop	0.69 (0.50–0.93)	0.02	1.00 (0.95–1.04)	0.86
2^nd^ intervention: Infectious diseases review	0.60 (0.44–0.80)	<0.01	1.02 (1.00–1.04)	0.09
3^rd^ intervention: infection control review and change in testing algorithm	1.12 (0.79–1.60)	0.52	0.98 (0.93–1.04)	0.54
Additional Variables	IRR (95% CI)		*P*-value	
COVID-19 Wave	0.79 (0.67–0.92)		<0.01	
Average length of hospitalization	0.70 (0.57–0.86)		<0.01	

*Pre-intervention*: January 2019–September 2019.

*1*^*st*^ *intervention*: October 2019–July 2020.

*2*^*nd*^ *intervention*: August 2020–February 2022.

*3*^*rd*^ *intervention*: March 2022–November 2022.

NHSN: National Healthcare Safety Network; IRR: Incidence rate ratio; CI: Confidence interval.

After the first intervention, the HO-CDI incidence decreased to 4.74 (95% CI: 3.00–7.11) reflecting a (52%) reduction (cIRR 0.48; 95% CI, 0.29–0.81). Unadjusted analysis indicated that there was a (43%) reduction in the admission CDI prevalence (cIRR: 0.57, 95% CI, 0.41–0.79) and a (45%) reduction in the total CDI prevalence (cIRR: 0.55, 95% CI, 0.42–0.73). However, the adjusted analysis revealed no significant changes (Supplemental Tables 1 and 2).

### Second intervention

From August 2020 to February 2022, the number of *C. difficile* tests per 1,000 PD decreased to 7.45 (95% CI, 6.93–7.99) and the test positivity after the second intervention increased to (19.8%) (*P* = 0.04). Notably, the test positivity increased when compared to the previous timeframe for tests completed between days 1 and 3 of hospitalization to (22.1%) (114/515, *P* = 0.03) (Table 1). *C. difficile* testing incidence decreased by (32%) (cIRR: 0.68, 95% CI, 0.61–0.75) and after adjustment, there was a (42%) reduction in *C. difficile* test incidence (aIRR: 0.58; 95% CI, 0.42–0.79) attributed to the second intervention (Table 3). The HO-CDI incidence decreased to 3.69 (95% CI, 2.61–5.07) reflecting a non-significant (22%) reduction in the HO-CDI rate (cIRR: 0.78; 95% CI, 0.46–1.31).

### Third intervention

From March 2022 to November 2022, the number of *C. difficile* tests decreased to 6.92 (95% CI: 6.21–7.69) per 1,000 patient days. The NAAT test positivity was similar between the third intervention and second intervention at (19.1% vs. 19.8%), respectively. However, accounting for specimens that were NAAT positive and toxin EIA positive, the test positivity was (4.9%) (*P* < 0.01) (Table 1).

While there was no impact on the *C. difficile* testing incidence attributed to the third intervention, accounting for only NAAT positive tests, the HO-CDI cases per 10,000 PD decreased to 1.40 (95% CI, 0.56–2.88) reflecting a 62% reduction in the HO-CDI cases per 10,000 PD (cIRR 0.38; 95% CI, 0.17–0.85). However, after accounting for tests that were NAAT positive and toxin EIA positive, there was a (95%) reduction in HO-CDI rate (cIRR: 0.05; 95% CI, 0.01–0.40). These changes also corresponded with a reduction in both the admission and total CDI prevalence (Table 3, Supplemental Tables 1 and 2).

## Discussion

In this single-center observational study, implementation of multiple *C. difficile* testing protocols at a small community-based hospital is feasible and could lead to a reduction in *C. difficile* testing as well as reported HO-CDI among hospitalized patients by the current NHSN definitions.^16^ The impact of these interventions was similar to results demonstrated in large academic healthcare centers which have demonstrated multiple methods to improve the NHSN-reported *C. difficile* metrics.^13,15^ However, smaller community-based hospitals often lack infection prevention resources when compared to their academic counterparts.^18,19^ Our hospital system was limited to three ID physicians and at most five ICPs overseeing these serial interventions making communication and coordination among the ICP staff and key stakeholders a critical. As noted at the beginning of the study period, our hospital had remarkably elevated *C. difficile* testing per 1,000 PD and HO-CDI per 10,000 PD which prompted our interventions. Likely our elevated *C. difficile* testing and HO-CDI incidence was due to multiple factors, but we hypothesis that over-testing in patients with a low *C. difficile* pre-test probability was the primary issue that required remediation.

Although NAAT is useful for the detection of *C. difficile*, the testing platform has led to a significant increase in CDI incidence.^7,11^ The increased sensitivity of NAAT leads to an increased risk of a hospital facing a financial penalty, especially based on the CMS value-incentive programs that measure HO-CDI. Through simple interventions such as ensuring the appropriateness of the *C. difficile* test order, our facility saw a dramatic decrease in the incidence rate of *C. difficile* tests ordered. The most notable impacts on *C. difficile* testing took place after introducing EMR guidance to healthcare providers assessing the appropriateness of *C. difficile* testing and after ID physician review of *C. difficile* tests ordered >3 days into a hospitalization. While these findings were similar to a study that took place in a large academic setting in which they noted a >26% reduction in *C. difficile* orders,^13^ as a smaller institution, our primary concern was providing a durable response. These data from our study indicate that we achieved a reduction in *C. difficile* testing and HO-CDI cases with a durable and sustainable over multiple years reinforced by the fact that our *C. difficile* testing and HO-CDI incidence continued to decline.

Notably, review of testing by ID physicians did not appear to impact the incidence of HO-CDI cases significantly. While previous studies have demonstrated that this is an effective measure to reduce HO-CDI cases,^15^ we hypothesize that given the size of our institution and the relative rarity of HO-CDI cases our crude analysis of HO-CDI was insufficient to capture a subtle decrease in HO-CDI. This hypothesis is supported by the fact that our second intervention led to a reduction in the number of *C. difficile* tests completed which were solely directed at tests that would have resulted in HO-CDI cases. Thus, the intervention was deemed successful by the institution by further reducing the number of inappropriate *C. difficile* tests completed.

The largest impact on the HO-CDI cases within our institution, as well as our admission and total CDI prevalence, can likely be attributed to changing our testing protocol to include toxin EIA testing with NAAT. Per the NHSN guidance, to qualify as a positive LabID event, the final *C. difficile* test in a series should be positive.^16^ Previous data has indicated that patients who are toxin EIA positive may be more likely to have a true infection whereas toxin EIA negative or have less severe disease.^20–22^ However, more recent data has shown that a large number of NAAT-positive and toxin EIA-negative patients still receive treatment.^23^ Thus, although reporting LabID event HO-CDI as cases which are NAAT positive and toxin positive after >72 hours may more appropriately identify true CDI cases, it does not factor in clinical assessment or when providers opt to treat a patient in toxin EIA negative cases.

The employment of rapid molecular diagnostics, such as PCR, has been increasingly used in improving the diagnosis of various infections.^24^ However, inappropriate testing can lead to unintended consequences, including overdiagnosis, inappropriate treatment, and increasing the risk of financial penalties.^8,9,25^ CDI diagnostic stewardship is part of a larger effort to improve ID stewardship.^25^

Our study is not without limitations. Our study took place during the COVID-19 pandemic which could have changed the typical hospital population or influenced testing habits of physicians due to increased antimicrobial usage. To account for the impact of COVID-19 we included a COVID-wave variable to account for regional COVID-19 hospitalization which noted COVID-19 was associated with a reduction in testing as well as a reduction in the prevalence of CDI within our patient population. Additionally, our study did not assess the appropriateness of the *C. difficile* test, nor the outcomes associated with the patients that were NAAT positive and toxin EIA positive or toxin EIA negative. Lastly, as HO-CDI is relatively rare, we were unable to construct a model to assess the impact our intervention had on HO-CDI rates.

In summary, these findings demonstrate that community-based hospitals can implement a series of changes that provide a durable reduction in the number of *C. difficile* tests and the reported HO-CDI cases.

## Supporting information

Wang et al. supplementary material Wang et al. supplementary material

## Supplementary material

For supplementary material accompanying this paper visit http://doi.org/10.1017/ash.2025.55. click here to view supplementary material
