# Three stages of laboratory stewardship in improving appropriate *Clostridioides difficile* testing in a community-based setting—CORRIGENDUM

**DOI:** 10.1017/ash.2025.10098

**Published:** 2025-08-08

**Authors:** Michael S. Wang, Gretchen Zimmerman, Theresa Klein, Bethany Stibbe, Monica Rykse, Samuel Ballard, Naveen Vijayam, Joe Brown, Khateeb Raza, Shannon Beckman, Andrew M. Skinner

In the initial publication of this article, author Theresa Klein’s first name was misspelled.^1^ The name has been corrected.
