# Supplementary material for: Three stages of laboratory stewardship in improving appropriate Clostridioides difficile testing in a community-based setting
Source: Antimicrob Steward Healthc Epidemiol. 2025 Mar 19;5(1):e81. doi: 10.1017/ash.2025.55 (PMC11920913; doi:10.1017/ash.2025.55)
Supplement: Wang et al. supplementary material [file S2732494X25000555sup001.docx]

**Supplementary Appendix for “Three Stages of Laboratory Stewardship in Improving Appropriate *Clostridioides difficile* Testing in a Community-Based Setting”**

**Table of Contents**

I. Supplemental Figure 1 2

Hard stop produced by the EMR when ordering a *C. difficile* test in patient who has not had 3 loose stools documented 2

II. Supplemental Figure 2 2

Hard stop produced by the EMR when ordering a *C. difficile* test in patient who is receiving a laxative 2

III. Supplemental Figure 3 2

Order that must be completed with instructions to contact ID physician in *C. difficile* tests ordered after 3 days 2

IV. Additional Statistical Analysis Methods 4

*Methods: Study Definitions* 4

*Methods: Negative binomial interrupted time series analysis* 4

*Methods: Poisson interrupted time series analysis* 6

*Results: Poisson interrupted time series analysis* 7

*Supplemental Table 1. Adjusted prevalence rate ratio of admission Clostridioides difficile infections per 100 admissions for implemented interventions* 8

*Supplemental Table 1. Adjusted prevalence rate ratio of total Clostridioides difficile infections per 100 admissions for implemented interventions* 9

**I. Supplemental Figure 1**

Hard stop produced by the EMR when ordering a *C. difficile* test in patient who has not had 3 loose stools documented


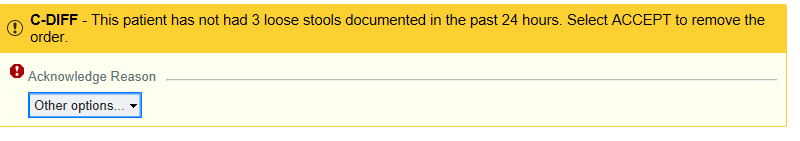


**II. Supplemental Figure 2**

Hard stop produced by the EMR when ordering a *C. difficile* test in patient who is receiving a laxative


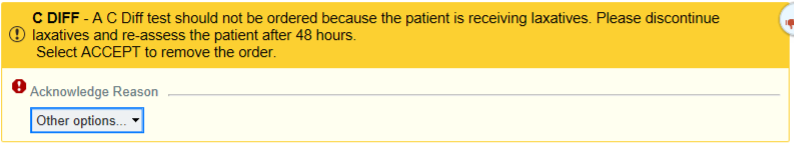


**III. Supplemental Figure 3**

Order that must be completed with instructions to contact ID physician in *C. difficile* tests ordered after 3 days


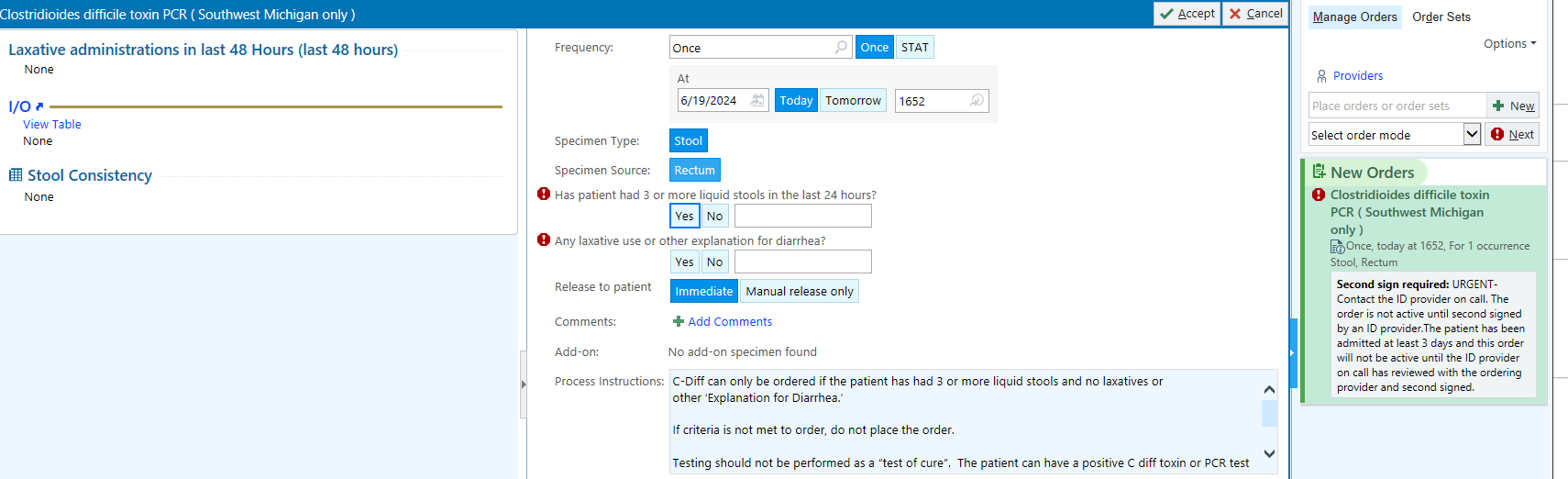


**IV. Additional Statistical Analysis Methods and Results**

*Methods: Negative binomial interrupted time series analysis*

We constructed a negative binomial model to complete an interrupted time series (ITS) analysis of the incidence of *C. difficile* tests ordered per 1000 patient days (PD) to determine the adjusted incidence rate ratio (aIRR). Prior to negative binomial model selection, a Poisson regression analysis was conducted with the variables described below. However, due to overdispersion, a negative binomial model was selected. The primary outcome assessed in the negative binomial interrupted model was the number of completed *C. difficile* tests per month per 1000 PD. The dependent variables selected *a priori* were 1) months prior to any intervention, 2) intervention 1 enacted, 3) time since intervention 1, 4) intervention 2 enacted, 5) time since intervention 2, 6) intervention 3 enacted, 7) time since intervention 3. Additionally, we created a dichotomous variable to account for COVID-19. To account for a lack of testing capacity, the months 3/2020 through 6/2020 were classified as being part of a “COVID-19 wave”. After which, any month that the state of Michigan reported a COVID-19 hospital admission rate of ≥ 30 admissions per 100,000 people was classified as being a “COVID-19 wave”. Additionally, previous data has demonstrated that prolonged hospitalization increases the odds of a patient undergoing a *C. difficile* test. As our study could not determine the patient days per each individual patient to calculate an average length of hospitalization, we utilized an indirect measurement average length of stay by dividing the monthly total PD by the number of monthly inpatient admission to account for any fluctuations in the average length of hospitalization. These variables were added in a stepwise fashion and compared by -2 log-likelihood comparison. Both the COVID-19 and monthly average length of stay variables were included in the final model.

*Methods: Poisson interrupted time series analysis*

We constructed a Poisson regression model to complete an ITS analysis of the prevalence of admission and total *C. difficile* infections (CDI) per 100 hospital admissions to determine the adjusted prevalence rate ratio (aPRR). Poisson models were constructed for each outcome and dispersion was confirmed to be <1.0 for each model. The outcomes assessed in the Poisson regression interrupted time series analysis were 1) admission CDI per month per 100 hospital admissions and 2) total CDI per month per 100 hospital admissions. The dependent variables selected *a priori* were 1) months prior to any intervention, 2) intervention 1 enacted, 3) time since intervention 1, 4) intervention 2 enacted, 5) time since intervention 2, 6) intervention 3 enacted, 7) time since intervention 3. Additionally, we assessed the COVID-19 and average length of hospital variables as previous described. These variables were added in a stepwise fashion and compared by -2 log-likelihood comparison. Only the COVID-19 variable was included in the final model.

*Results: Poisson interrupted time series analysis*

The Poisson ITS analysis of admission CDI prevalence revealed that the 1^st^ and 2^nd^ interventions contributed to a non-significant decrease in the admission CDI by 42% (aPRR: 0.58; 95% CI: 0.30 – 1.10) and 42% (aPRR: 0.58; 95% CI: 0.28 – 1.23), respectively. In the months following these interventions, these interventions had no impact on the admission CDI per 100 admissions (Supplemental table 1). The 3^rd^ intervention accounted for a non-significant 21% reduction in admission CDI prevalence (aPRR: 0.79; 95% CI: 0.39 – 1.56) when accounting for NAAT positive tests. However, when accounting for toxin positive events, the 3^rd^ intervention accounted for a 75% reduction in admission CDI prevalence (aPRR: 0.25; 95% CI: 0.08 – 0.71). However, in the months following the intervention, there was no additional impact on the admission CDI prevalence.

The Poisson ITS analysis of total CDI prevalence revealed that the 1^st^ and 2^nd^ interventions contributed to a non-significant decrease in the total CDI prevalence by 36% (aPRR: 0.64; 95% CI: 0.38 – 1.09) and 19% (aPRR: 0.81; 95% CI: 0.43 – 1.55), respectively. In the months following these interventions, these interventions had no impact on the total CDI per 100 admissions (Supplemental table 1). The 3^rd^ intervention accounted for a non-significant 22% reduction in total CDI prevalence (aPRR: 0.77; 95% CI: 0.40 – 1.43) when accounting for NAAT positive tests. However, when accounting for toxin positive events, the 3^rd^ intervention accounted for a 76% reduction in total CDI prevalence (aPRR: 0.24; 95% CI: 0.08 – 0.64). However, in the months following the intervention, there was no additional impact on the total CDI prevalence.

*Supplemental Table 1. Adjusted prevalence rate ratio of admission Clostridioides difficile infections per 100 admissions for implemented interventions*

|  | *C. difficile* tests per 1000 patient-days | | | |
| --- | --- | --- | --- | --- |
|  | Immediate IRR (95% CI) | p-value | IRR change per month (95% CI) | p-value |
| Pre-intervention |  |  | 1.00 (0.93 – 1.08) | 0.99 |
| 1^st^ intervention: Electronic medical record hard stop | 0.58 (0.30 – 1.10) | 0.10 | 1.04 (0.91 – 1.18) | 0.58 |
| 2^nd^ intervention: Infectious diseases review | 0.58 (0.28 – 1.23) | 0.15 | 1.01 (0.92 – 1.12) | 0.81 |
| 3^rd^ intervention: infection control review and change in testing algorithm | 0.79 (0.39 – 1.56)  /  0.25 (0.08 – 0.71) | 0.50  /  0.01 | 0.92 (0.83 – 1.02)  /  0.89 (0.73 – 1.07) | 0.12  /  0.22 |
|  |  |  |  |  |
| Additional Variables | IRR (95% CI) | | p-value | |
| COVID-19 Wave | 0.61 (0.44 – 0.85)  /  0.61 (0.42 – 0.90) | | <0.01  /  0.01 | |
| Pre-intervention: January 2019 – September 2019  1^st^ intervention: October 2019 – July 2020  2^nd^ intervention: August 2020 – February 2022  3^rd^ intervention: March 2022 – November 2022  NHSN: National Healthcare Safety Network; IRR: Incidence rate ratio; CI: Confidence interval; | | | | |

*Supplemental Table 1. Adjusted prevalence rate ratio of total Clostridioides difficile infections per 100 admissions for implemented interventions*

|  | *C. difficile* tests per 1000 patient-days | | | |
| --- | --- | --- | --- | --- |
|  | Immediate IRR (95% CI) | p-value | IRR change per month (95% CI) | p-value |
| Pre-intervention |  |  | 1.02 (0.95 – 1.09) | 0.63 |
| 1^st^ intervention: Electronic medical record hard stop | 0.64 (0.38 – 1.09) | 0.10 | 0.97 (0.87 – 1.08) | 0.63 |
| 2^nd^ intervention: Infectious diseases review | 0.81 (0.43 – 1.55) | 0.52 | 1.05 (0.96 – 1.14) | 0.30 |
| 3^rd^ intervention: infection control review and change in testing algorithm | 0.77 (0.40 – 1.43)  /  0.24 (0.08 – 0.64) | 0.41  /  <0.01 | 0.94 (0.85 – 1.03)  /  0.89 (0.73 – 1.07) | 0.17  /  0.23 |
|  |  |  |  |  |
| Additional Variables | IRR (95% CI) | | p-value | |
| COVID-19 Wave | 0.69 (0.51 – 0.92)  /  0.67 (0.49 – 0.94) | | 0.01  /  0.02 | |
| Pre-intervention: January 2019 – September 2019  1^st^ intervention: October 2019 – July 2020  2^nd^ intervention: August 2020 – February 2022  3^rd^ intervention: March 2022 – November 2022  NHSN: National Healthcare Safety Network; IRR: Incidence rate ratio; CI: Confidence interval; | | | | |
